# Case of Bundibugyo Virus Disease in Goma, Democratic Republic of the Congo with Lack of Transmission to Breastfeeding Infant

**DOI:** 10.4269/ajtmh.26-0480

**Published:** 2026-10-13

**Authors:** Felicien M. Maisha, Kahatwa K. Serge, Kambale Kasonia, Zebedée Luhavo Kakule, Jayakrishnan Ajayakumar, John A. Lednicky, Andrew J. Curtis, J. Glenn Morris

**Affiliations:** ^1^ HEAL Africa Hospital, Goma, Democratic Republic of the Congo;; ^2^ Department of Sociology, University of Goma, Goma, Democratic Republic of the Congo;; ^3^ Emerging Pathogens Institute, University of Florida, Gainesville, Florida, USA;; ^4^ Department of Internal Medicine, University of Goma, Goma, Democratic Republic of the Congo;; ^5^ Department of Internal Medicine, Universite Libre des Pays de Grands Lacs, Goma, Democratic Republic of the Congo;; ^6^ Department of Clinical Research, Faculty of Infectious and Tropical Diseases, London School of Hygiene and Tropical Medicine, London, United Kingdom;; ^7^ Department of Population and Quantitative Health Sciences, Case Western Reserve University School of Medicine, Cleveland, Ohio, USA;; ^8^ Department of Environmental and Global Health, College of Public Health and Health Professions, University of Florida, Gainesville, Florida, USA

## Abstract

Disease due to Bundibugyo virus (BDBV), an *Orthoebolavirus* species, reemerged in April 2026, in Ituri Province, Democratic Republic of the Congo. A 35-year-old woman transported the body of a possible BDBV-infected patient from Bunia in Ituri Province to Goma for burial; she was accompanied by an 8-month-old breastfeeding child. Six days after arrival in Goma, she noted onset of fever and malaise and was empirically started on therapy for malaria; 4 days later she developed bloody diarrhea and a bloody vaginal discharge and was started on metronidazole for possible amebic dysentery. When her condition worsened she was admitted to hospital in Goma, where a blood sample was positive by real-time polymerase chain reaction (RT-PCR) test for BDBV, with a second positive test on hospital day 7. By day 14 of hospitalization fever and major symptoms had resolved and repeat RT-PCR tests × 2 were negative. Her child did not show evidence of infection, and no family members or health care workers at HEAL Africa Hospital developed illness.

## INTRODUCTION

Ebolavirus disease, first reported in 1976, is a viral hemorrhagic fever characterized by systemic inflammation, endothelial dysfunction, coagulopathy, and multiorgan system failure, with a high associated mortality rate.^1^^–^^3^ Ebolaviruses are enveloped, negative-sense RNA viruses within the genus *Orthoebolavirus*.^4^ Three species within this genus have been linked with outbreaks of human disease: the “original” ebolavirus from the Democratic Republic of the Congo (DRC), formerly Zaire (*Orthoebolavirus zairense* [EBOV]); Sudan virus (*Orthoebolavirus sudanense* [SUDV]); and Bundibugyo virus (*Orthoebolavirus bundibugyoense* [BDBV]).^1^^,^^4^ EBOV has been responsible for the most reported cases, including the major outbreak that occurred in West Africa in 2014–2016 with more than 28,600 cases, and may have a mortality rate of up to 90% among patients who do not receive treatment.^2^

Bundibugyo virus was first identified in an outbreak (131 suspect cases) that occurred in Bundibugyo District in western Uganda in August 2007 (Figure 1).^3^ The overall fatality rate among suspect cases was 34%, substantially lower than the rates reported for other *Orthoebolavirus* species.^1^^–^^3^ In an associated epidemiological investigation, infection was significantly associated with handling of bodies of deceased patients without appropriate protections.^3^ A second, smaller outbreak of BDBV disease was identified in 2012 in Isiro in Orientale Province of the DRC; 62 cases were reported, with a mortality rate of 55%.^5^ In 2026, BDBV disease was again identified in the DRC, this time in Ituri Province with a focus in Bunia (Figure 1). The putative index case became ill on April 24, 2026, with subsequent spread of the infection in Bunia and adjacent areas.^2^^,^^6^ On laboratory testing on April 30, clinical samples from these patients were negative for EBOV on a pathogen-specific GeneXpert assay. As case numbers continued to increase, samples were sent to the Institut National pour la Recherche Biomedicale (INRB) in Kinshasa, where they tested positive for BDBV on May 14, based on results of the pan-Orthoebolavirus RADI-ONE test (KH Medical; Pyeongtaek-si, South Korea) combined with sequence analysis.^7^ On May 17, the WHO declared the BDBV outbreak a Public Health Emergency of International Concern.^8^

**Figure 1.**
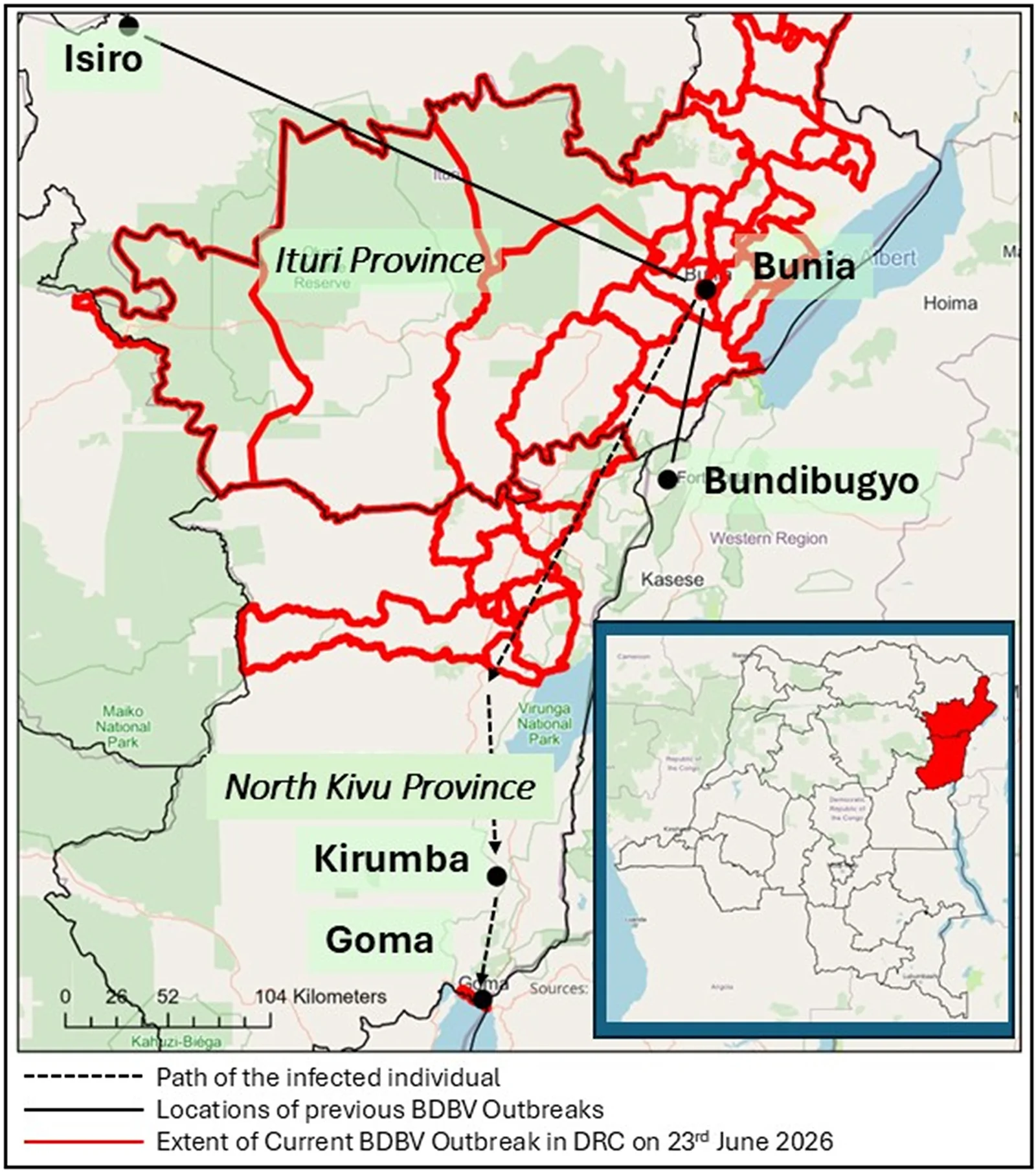
Map of DRC, showing areas of current and prior BDBV infection. Bundibugyo, in far western Uganda, was the site of the original BDBV outbreak in 2007. The 2012 outbreak occurred in Isiro in the DRC’s Orientale Province. The current outbreak is centered in Bunia in what is now Ituri Province. The route taken by Patient B to take the body of Patient A to Goma is shown as a dotted line on the map. BDBV = Bundibugyo virus; DRC = Democratic Republic of the Congo.

## CASE REPORT

In January 2026, a resident of Goma, DRC (Patient A) traveled to Bunia on business. She subsequently became ill and died on May 1 after presenting to the hospital in Bunia with fever, fatigue, and dyspnea. At the hospital, her oxygen saturation was low and blood pressure was undetectable, consistent with a shock syndrome; no hemorrhagic symptoms were reported. Testing for BDBV was not available at the time of her death. Her sister-in-law (Patient B), age 35 years, who was a local businesswoman in Bunia with prior nursing training, helped in preparation of the body (including washing and dressing the body and preparing it for viewing) and was asked to accompany the body back to Goma, DRC, for burial, a road distance of approximately 550 kilometers (Figure 1). Patient B traveled with another relative and her 8-month and 2-year–old children; her 8-month-old child was still breastfeeding. On May 2, the body, in a body bag, was transported by car from Bunia to Kirumba, where it was left overnight in a local hospital morgue; for the trip the body bag was placed on the front seat next to the driver, with the other passengers riding in the back seat. On May 3, the body was brought to a hospital morgue in Goma, where it was kept until May 6, when burial took place.

On May 9, Patient B began to experience fever and myalgia and was treated at home by a family member who was a physician. Because of concerns about malaria, which is endemic in Bunia, the patient was started empirically on artesunate. On May 13, she noted onset of diarrhea with bloody stools and vaginal bleeding; a stool test was positive for ameba, and she started taking metronidazole. That same day she stopped breastfeeding because she was weak and had difficulty holding the baby. Her condition continued to worsen, and, because news had just been released about the presence of BDBV infections in Bunia, on May 15 she was taken for further evaluation to HEAL Africa Hospital, a major tertiary-referral Congolese-led teaching hospital in Goma, where she was admitted.

On hospital admission (May 15) the patient appeared to be in distress, with a temperature of 38.5°C and complaints of malaise, vomiting, diarrhea, photophobia, and cough. On exam, she had an inflamed conjunctiva, with dry oral mucosa; pulmonary and cardiac exams were unremarkable, and abdomen was soft and nontender. Her hematocrit was 31%, with 235,000 platelets and an initial thick blood film consistent with malaria. A blood sample was also sent to the INRB laboratory in Goma for testing for BDBV using the RealStar Bundibugyo Virus RT-PCR Kit™ (Altona Diagnostics; Hamburg, Germany). The differential diagnosis at the time of admission included shigellosis, salmonellosis (including typhoid), malaria, and ebolavirus disease. Therapy with artesunate was continued; the patient was given antibiotics with activity against *Shigella* and *Salmonella*/typhoid (Ornidox^®^ [ofloxacin and ornidazole]); and intravenous fluids, antiemetics and antipyretics were administered.

The following day INRB reported that the patient’s sample was positive for BDBV; this was the first BDBV case reported in Goma after onset of the outbreak. Patient B was isolated in the hospital and materials, equipment, and household premises were decontaminated, in keeping with the recommendations of WHO and the DRC Ministere de la Sante Publique, Hygiene et Prevoyance Sociale (MSP). Known patient contacts were quarantined, and the patient stopped breastfeeding. For hospital employees caring for the patient, full isolation suits were used (Figure 2), with careful disposal of contaminated waste. The patient was managed in keeping with the “Protocole de Traitement de la maladie a viral Ebola; simple et compliquée,” prepared by the DRC MSP and informed by the WHO related protocol. Vomiting and loose stools subsided by hospital day 5 (May 20). However, a repeat test for BDBV by INRB on May 22 remained positive. The patient’s fever subsided by day 14 of hospitalization (May 29) (Figure 3), with resolution of major symptoms. A repeat test for BDBV on May 29 was negative, with a second negative test obtained on June 2. The patient was discharged from hospital on June 9.

**Figure 2.**
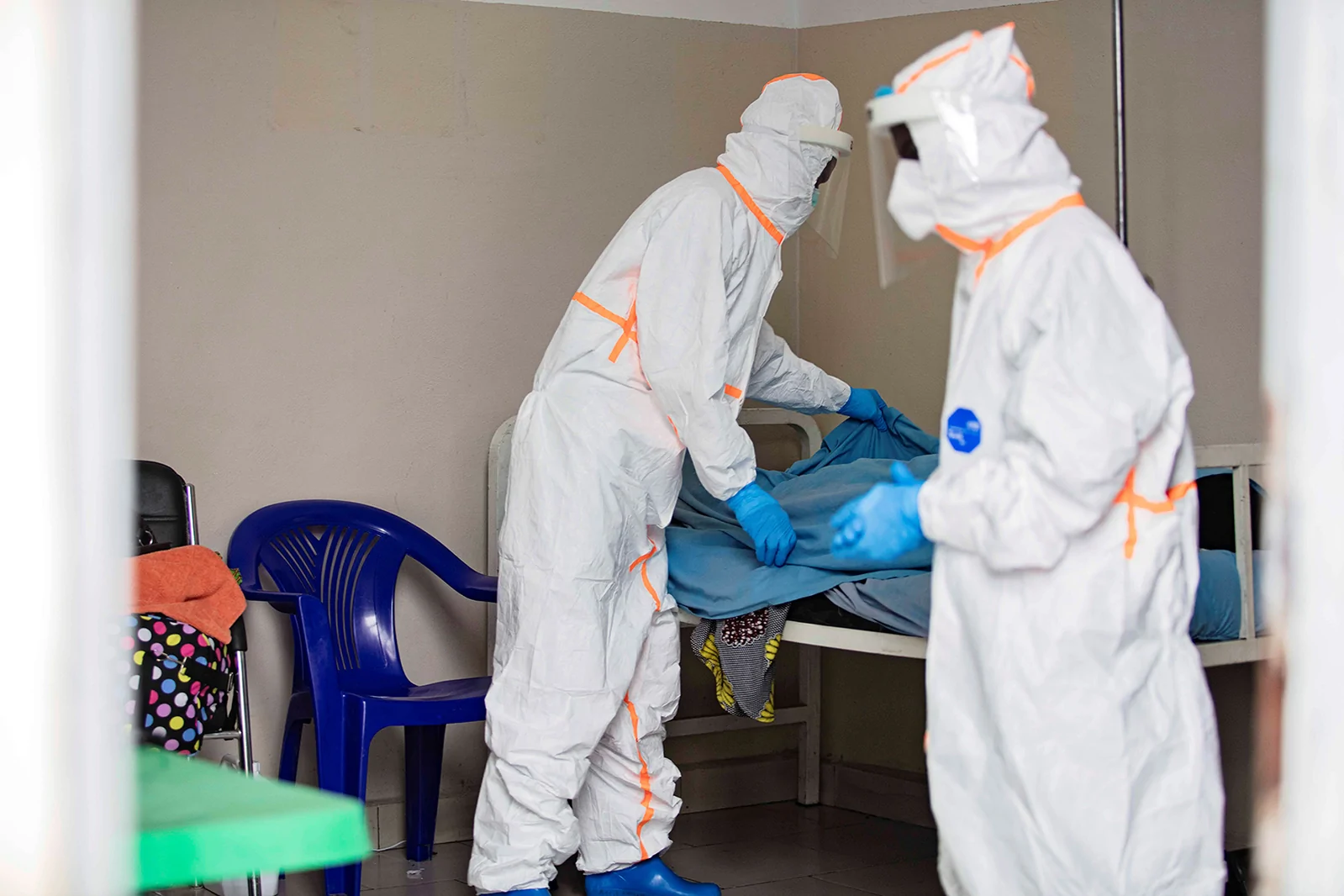
Nursing personnel caring for Patient B at HEAL Africa Hospital, Goma.

**Figure 3.**
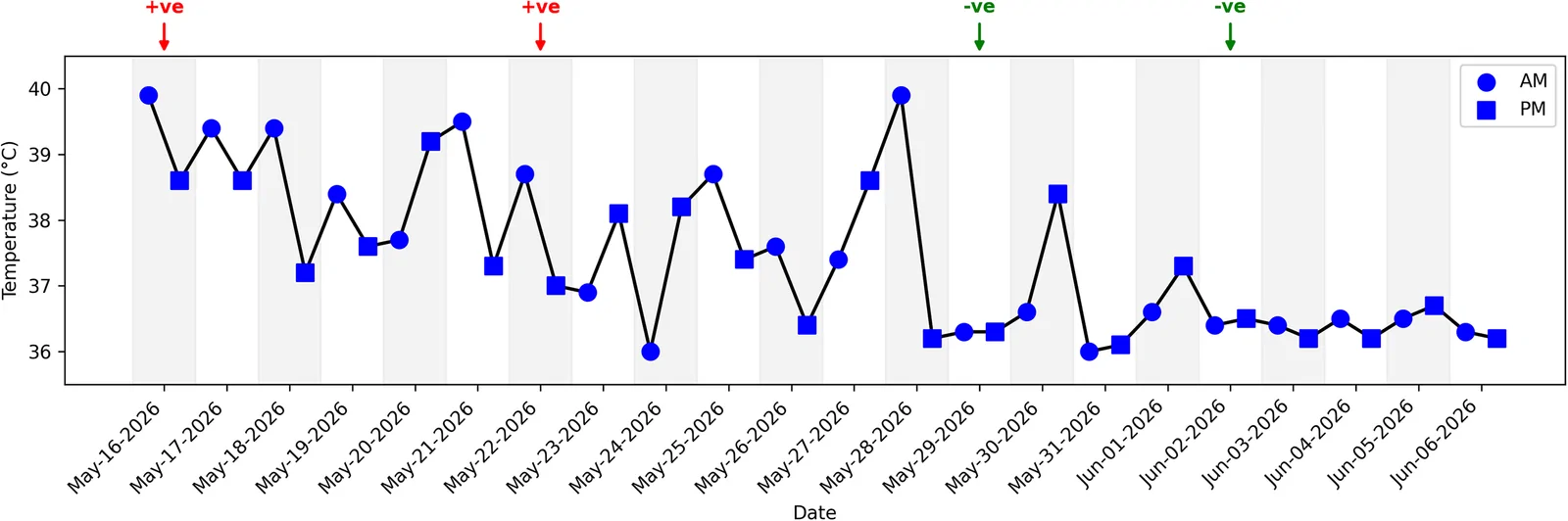
Fever chart, Patient B, from hospital admission to discharge. Dates and results of real-time polymerase chain reaction testing are indicated above the chart.

In the 3-week period after discharge of Patient B from HEAL Africa Hospital, there were no reports of illness or infections among hospital staff. The patient’s 8-month-old child who was breastfeeding showed no signs of illness.

## DISCUSSION

Symptoms of our patient were similar to those reported by Akilimali et al. in their study of early BDBV disease cases in the Bunia region, in which the most common symptoms among patients with laboratory-confirmed BDBV infection (*N* = 405) included fever (74%), vomiting (67%), and diarrhea (67%).^9^ Our case patient also had conjunctival inflammation (reported by 1.7% of patients in the Akilimali study) and cough (10% of patients). Given the lack of specificity of these symptoms, it is not surprising that the initial differential diagnosis for our patient was wide-ranging and resulted in treatment of other diseases known to be common in the region. Hemorrhage is often regarded as a key diagnostic indicator for ebolavirus infection; however, in the Akilimali study, only 10% of BDBV patients reported hemorrhagic signs or bleeding and rates of hemorrhage were not significantly different between patients who were RT-PCR positive for BDBV and those who were negative.^1^^,^^2^^,^^9^ The overall reported mortality rate reported by the WHO among confirmed BDBV cases as of July 2, 2026, was 32%. Although the reported rates of hemorrhage and mortality need to be confirmed with further data, results are consistent with the hypothesis that BDBV illness is less severe than that seen with other *Orthoebolavirus* species, with reduced mortality rates in settings in which optimized supportive care can be provided.^1^^,^^9^^–^^11^

Contact with body fluids from infected patients has consistently been cited as the primary mode of transmission for ebolaviruses.^1^^,^^2^ Factors determining infectivity of individual patients and risk of transmission are less well understood, with virtually no data available for BDBV.^12^ Given the strong epidemiological association noted in the 2007 BDBV outbreak between infection and handling of patient bodies,^3^ there is a reasonable likelihood that Patient B was infected while preparing Patient A’s body. However, there was no evidence of subsequent transmission of infection from Patient B to other members of her family. In particular, her 8-month-old child, whom she was breastfeeding until shortly before hospital admission, was not symptomatic and did not appear to be infected. Data on risk of transmission of ebolaviruses by breast milk are limited.^13^ A 2020 systematic review included eight studies with a total of 25 infants who were breastfed by women infected with EBOV and SUDV.^14^ Although 17 of these infants developed ebolavirus disease, eight remained healthy, and those who became ill had a number of potential exposures to the virus other than breast milk. Identification of ebolavirus in breast milk by RT-PCR has been reported, but RT-PCR results do not necessarily indicate that viable infectious virus is present; this generally requires virus culture.^15^ We also saw no evidence of BDBV transmission to hospital staff. The HEAL Africa Hospital in Goma is recognized as a major referral center for this region of the DRC, with good staff training and equipment. With appropriate infection prevention and control training and equipment, it is clearly possible to minimize risk of transmission within local hospitals.

Surveillance data have documented rapid, ongoing spread of BDBV infections in Ituri Province and surrounding areas. Our case underscores the need for more in-depth study of BDBV transmission pathways and/or factors driving infectivity, given the number of persons who did not become ill despite exposure to the body of Patient A and/or to Patient B. Returning to the beginning of the outbreak, there are also questions about source of the virus and virus reservoirs. In a recently reported analysis of whole viral genome data,^7^ isolates from the 2007, 2012, and current 2026 outbreak each form distinct, well-supported phylogenetic clusters. For each of the three outbreaks, findings are consistent with an initial zoonotic “spillover” event, followed by community-based person-to-person transmission; data do not support resurgence or transfer of isolates from one outbreak to the next. This evidence, in turn, raises questions about the existence of BDBV animal reservoirs such as bats or nonhuman primates and their role in transmission.^2^^,^^3^^,^^16^ Further studies of transmission pathways, asymptomatic infection and carriage, and risk factors for infection will be critical for BDBV control, in conjunction with rapid development of specific vaccines and therapies for this emergent pathogen.^2^^,^^10^^,^^17^^–^^19^
